# The role of von Willebrand factor in breast cancer metastasis

**DOI:** 10.1016/j.tranon.2021.101033

**Published:** 2021-02-08

**Authors:** Chia Yin Goh, Sean Patmore, Albert Smolenski, Jane Howard, Shane Evans, Jamie O'Sullivan, Amanda McCann

**Affiliations:** a UCD Conway Institute of Biomolecular and Biomedical Research, University College Dublin, Belfield, Dublin, Dublin 4, Ireland; b UCD School of Medicine, College of Health and Agricultural Sciences (CHAS), University College Dublin, Belfield, Dublin, Dublin 4, Ireland; c Irish Centre for Vascular Biology, School of Pharmacy and Biomolecular Sciences, Royal College of Surgeons in Ireland, Dublin, Dublin 2, Ireland

**Keywords:** VWF, Disseminated disease, Angiogenesis, Hypercoagulopathy, Tumour cell-induced platelet aggregation

## Abstract

Breast cancer is the most common female cancer globally, with approximately 12% of patients eventually developing metastatic disease. Critically, limited effective treatment options exist for metastatic breast cancer. Recently, von Willebrand factor (VWF), a haemostatic plasma glycoprotein, has been shown to play an important role in tumour progression and metastasis. In breast cancer, a significant rise in the plasma levels of VWF has been reported in patients with malignant disease compared to benign conditions and healthy controls, with an even greater increase seen in patients with disseminated disease. Direct interactions between VWF, tumour cells, platelets and endothelial cells may promote haematogenous dissemination and thus the formation of metastatic foci. Intriguingly, patients with metastatic disease have unusually large VWF multimers. This observation has been proposed to be a result of a dysfunctional or deficiency of VWF-cleaving protease activity, ADAMTS-13 activity, which may then regulate the platelet-tumour adhesive interactions in the metastatic process. In this review, we provide an overview of VWF in orchestrating the pathological process of breast cancer dissemination, and provide supporting evidence of the role of VWF in mediating metastatic breast cancer.

## Highlights

• VWF plays an important role in breast tumour progression and metastasis.
• Patients with metastatic breast cancer have significantly elevated plasma VWF.
• Increased levels of highly adhesive VWF may regulate platelet-tumour interactions.
• VWF may protect disseminated tumour cells from chemotherapy.

## Graphical abstract

**Figure:**
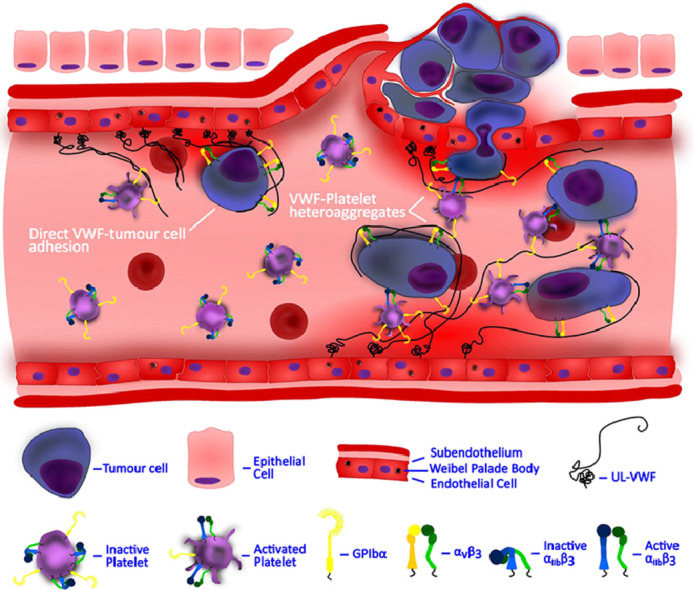

## Introduction

Breast cancer is the most common female malignancy with 523,000 cases reported in Europe in 2018 [1]. Although huge advancements have been made in the treatment of breast cancer, the prevention of tumour progression and metastasis remains a clinical challenge. Despite developments in frontline therapy, about 30% of the patients with breast cancer do not respond to treatment, and approximately 12% eventually develop metastatic disease [2,3]. Unfortunately, metastatic breast cancer is associated with a poor prognosis and a low 5-year-survival rate of 26% [2]. This warrants the need for a reliable biomarker that would enable early detection of metastasis, and the discovery of more effective anti-metastatic therapies.

During the metastatic process of cancers, primary tumour cells dislodge from the tumour mass and intravasate across the endothelium to enter blood vessels. Tumour cells then travel systemically in the circulation, and extravasate to secondary sites, establishing micro- and macro-metastasis. The interactions between cancer cells and endothelial cells are crucial in driving metastasis [4]. In the absence of endothelial damage or activation, the vessel endothelial cells remain quiescent [5,6]. However, dysfunctional endothelium, including inflammation and activation of the vessels, triggers the upregulation of adhesive molecules, secretion of growth factors and cytokines, and alters vascular permeability. All of these contribute to cancer metastasis by facilitating the adhesion of tumour cells to the endothelium, and promoting transendothelial migration [5,6]. The activation of endothelial cells also triggers the secretion of VWF multimers into the lumen of vessel as well as the subendothelial matrix [7]. Critically, these VWF multimers serve as a molecular bridge, facilitating the adhesion and aggregation of platelets and tumour cells along the endothelium, promoting transendothelial migration and subsequently cancer dissemination [6, 8]. Emerging evidence now also suggests that platelet-decorated VWF multimers tether immune cells, including neutrophils and monocytes, promoting diapedesis and migration of leukocytes to sites of inflammation [9,10].

Significant interplay exists between coagulation and cancer, first described by Trousseau in the 1860′s [11]. Trousseau's syndrome or cancer-associated thrombosis is in fact the second leading cause of death in cancer patients, with the risk of venous thromboembolism (VTE) between 4- to 7-fold higher in patients with cancer than in those without cancer [12,13]. This risk is highest in patients with advanced metastatic cancer [12]. Intriguingly, there is increasing evidence suggesting that the coagulation pathways and haemostatic proteins are not mere bystanders in the process of cancer progression. In fact, the blood coagulation system facilitates tumour progression and dissemination, while its inhibition through anticoagulation has been shown to significantly attenuate the metastatic potential of cancer cells in several animal models [14], [15], [16], [17]. Importantly, VWF is a major determinant of VTE in cancer patients. Emerging evidence also suggests that VWF may orchestrate the metastatic process independent of its established haemostatic functions [18]. This indicates the complex intertwined relationship between coagulation and metastasis, whereby coagulation activation not only promotes thrombosis in cancer patients but may also contribute directly to cancer progression.

## Structure, physiological functions and proteases of VWF

VWF is produced in endothelial cells and megakaryocytes. Following its biosynthesis, VWF is stored in the α-granules of megakaryocytes/ platelets, as well as the Weibel-Palade bodies (WPB; storage granules in endothelial cells) [19]. Interestingly, recent evidence suggests that VWF is also synthesised and released by cancer cells [20].

**Fig. 1:**
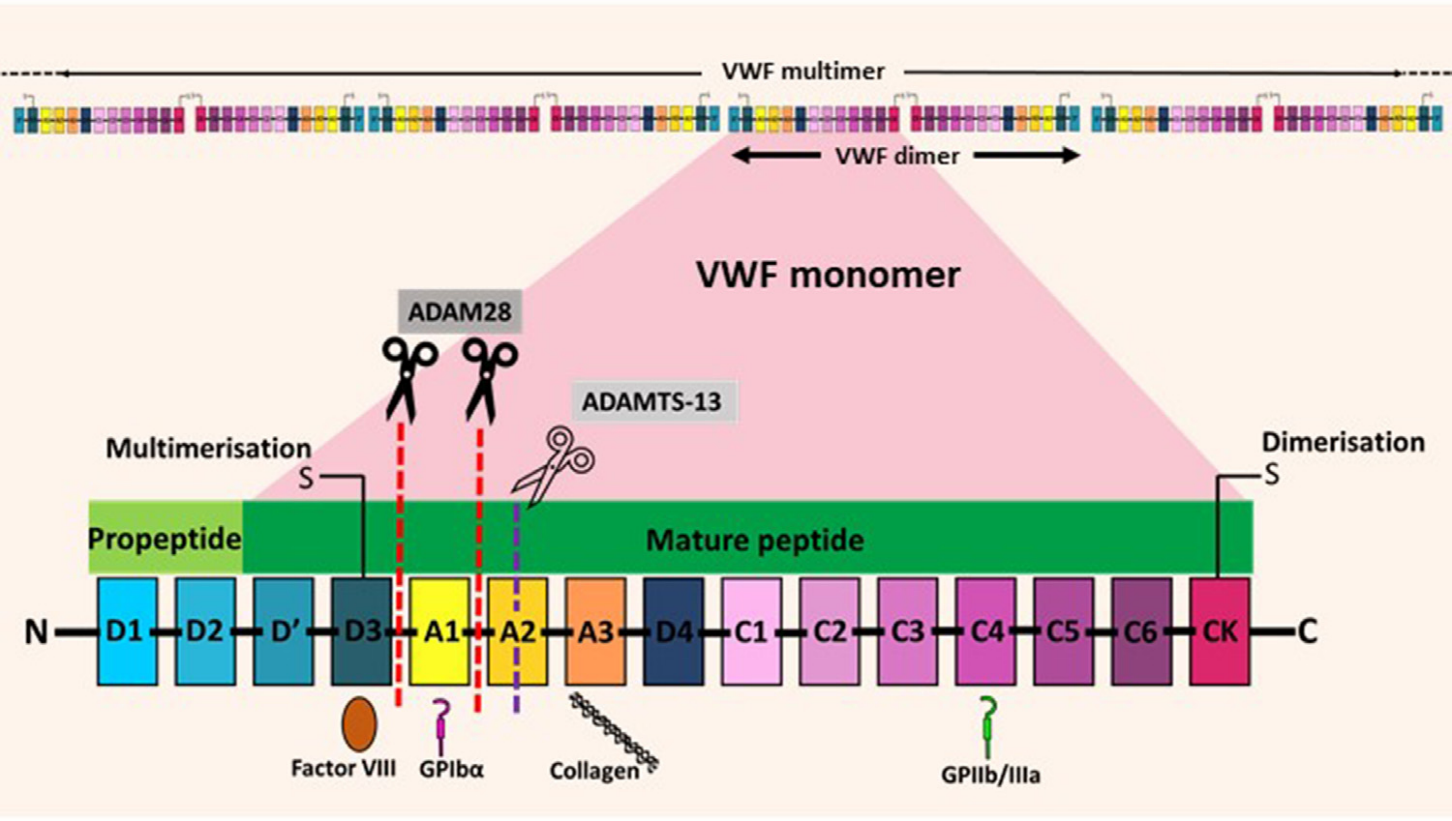
**VWF as a complex multimeric plasma glycoprotein.** The D'D3 domain is essential for the formation of VWF multimers, and the CK domain is responsible for dimerisation via disulphide linkages [25, 27, 28]. The protease ADAM28 cleaves the linking regions of D3-A1 and A1-A2 domains [30], whereas ADAMTS-13 cleaves VWF at the A2 domain [29, 31]. Some of the well characterised ligands of VWF contributing to its haemostatic function include coagulation factor VIII (binds to D3 domain) [21], GPIbα (binds to A1 domain) [22], collagen type III (binds to A3 domain) [23] and GPIIb/IIIa (binds to C4 domain) [24].

VWF is a complex multi-domain structure that interacts with a variety of ligands, including collagen, coagulation factor VIII, as well as several endothelial and platelet integrins, P-selectin, αvβ3, GPIIb/IIIa and GPIbα [21], [22], [23], [24]. VWF is a large multimeric protein comprising multiple monomers (∼270 kDa), with the size of multimers in the plasma found to be up to 20,000 kDa. Importantly, the multimerisation of VWF is a critical determinant of its functional activity [9,25]. Recent reassessment of the mosaic architecture of VWF has led to the proposal of its repeated domain structures (Fig. 1) in the order of D1-D2-D′-D3-A1-A2-A3-D4-C1-C2-C3-C4-C5-C6-CK [25,26]. Notably, (i) the N-terminal D’-D3 domains contain disulphide linkages for multimer formation, and also serve as the binding region for coagulation factor VIII, (ii) the central A-domains are responsible for much of its adhesive functions, which regulate the binding to collagens and platelets, and (iii) the C-terminal cystine knot (CK) domain is important for VWF dimerisation [25,27,28]. Under physiological conditions, VWF multimerisation is regulated by specific proteases, ADAMTS-13 (A disintegrin and metalloproteinase with a thrombospondin type 1 motif, member 13), which cleaves VWF at a unique site within the A2 domain, converting highly active high molecular weight multimers into less active lower molecular weight forms [29]. More recently, an additional member of the ADAMs protease family, ADAM28, has also been shown to cleave VWF [30]. ADAM28 appears to be of particular importance in the context of cancer cell biology, since it is highly expressed by tumour cells [30].

Biologically, VWF plays a pivotal role in haemostasis [32]. During a blood vessel injury, the subendothelium is exposed. This enables circulating VWF to bind to collagens, leading to shear stress-induced unfolding of the A1 domain and tethering of flowing platelets via platelet receptors, glycoproteins Ibα (GPIbα) and IIb/IIIa (GPIIb/IIIa; also known as α_IIβ_β_3_ integrin), thus forming a platelet thrombus at the site of injury [25]. GPIbα is mainly responsible for platelet-vessel wall adhesion, whereas GPIIb/IIIa participates in both platelet-vessel wall adhesion and platelet crosstalk [31]. Subsequently, the platelets adhere to fibrins, a process mediated by the C domains of VWF under high shear stress conditions [33]. VWF also serves as a carrier of the coagulation factor VIII, which is essential for normal haemostasis [31].

Recent research has led to the discovery of additional non-haemostatic functions of VWF, including smooth muscle cell proliferation [34,35], immune response [36], angiogenesis [32, [37], [38], [39]] and cancer metastasis [9,18,20].

### ADAMTS-13 protease: a disintegrin and metalloproteinase with a thrombospondin type 1 motif, member 13

The size of VWF multimers, and thus its haemostatic activity, is critically regulated by ADAMTS-13 that cleaves VWF into smaller multimers under shear forces in flowing blood [25]. ADAMTS-13 specifically cleaves within the A2 domain of VWF, at position Tyr1605-Met1606 [29, 31]. The unfolding of VWF and exposure of the cryptic cleavage site are the prerequisites of ADAMTS-13 proteolytic activity; thus, these processes are dependent on the shear stress in the circulation [31]. After proteolysis, the ultra-large and highly haemostatically active VWF is reduced into smaller and less active forms [31, 40]. Deficiency or dysfunction in ADAMTS-13 results in the life threatening microangiopathy termed thrombotic thrombocytopenic purpura (TTP) [41]. TTP is characterised by the unregulated accumulation of large adhesive VWF multimers and consumption of platelets in platelet-rich microthrombi within the vasculature.

### ADAM28 protease: a disintegrin and metalloproteinase 28

Unlike ADAMTS-13 which only cleaves VWF under conditions of shear stress that induce unfolding of the A2 domain, ADAM28 also cleaves native VWF. ADAM28 targets the linker regions of D3-A1 and A1-A2 domains (Fig. 1) [30]. Little is known about the physiological roles of ADAM28 on VWF. However, the role of ADAM28 expression is implicated in cancer metastasis. For example, ADAM28 expression by tumour cells enhances lung metastasis in various cancers including breast and renal cell carcinoma [30], and its inhibition has been shown to suppress non-small cell lung cancer (NSCLC) metastasis [42].

## VWF adhesion and interactions with breast cancer cells

With regards to VWF expression in different subtypes of breast cancer, it has been reported that patients with invasive lobular carcinoma (ILC) have higher VWF RNA expression than patients with invasive ductal carcinoma (IDC) and other histology presentations [43]. In addition, VWF and PTEN were also found to share nine co-occurrent alterations in ILC, possibly working in tandem to promote tumour progression [43]. Another study has shown that VWF tumour mRNA levels correlated with its VWF serum protein levels in patients with HER2-negative breast cancer, suggesting that VWF might be produced by tumour cells with an outflow to the systemic circulation [44].

Growing evidence demonstrates that tumour cells not only induce the release of VWF multimers from endothelial cells, but also utilise VWF to adhere to the endothelium [45]. Furthermore, the VWF multimers may serve as a bridging platform that tethers platelets and tumour cells to form heterotypic aggregates, promoting cancer metastasis by facilitating transendothelial migration across the blood vessel wall [46]. The role of the platelet-tumour aggregates in metastasis are detailed in the subsequent sections of this review. The aggregates may also form a platelet “cloak” that shields tumour cells from immune surveillance and natural killer cell-mediated cytolysis [47]. Moreover, MCF-7 breast cancer cells have been reported to express pseudo-GPIbα receptors on their surface which may facilitate direct interactions with VWF, independent of platelets [48]. In support of this, treatment of MCF-7 and MDA-MB-231 breast cancer cells using GPIbα antibodies, not only reduced platelet-tumour cell interactions, but also attenuated the adhesion of tumour cells to endothelial cells in vitro [49].

Integrin expression is one of the important contributors to the increased metastatic potential of tumour cells. It is known that VWF binds to tumour cells via GPIIb/IIIa receptor and its hemi-identical twin, αvβ3 integrin [30, [50], [51], [52]]. The αvβ3 integrin shares the same β subunit as GPIIb/IIIa receptor, and its α subunit shares 40% homology with αIIb [53]. A static cell adhesion model demonstrated that binding of VWF to B16-BL6 melanoma cells was mediated by αvβ3 integrin expressed on the tumour cell surface [52]. Moreover, under condition of shear stress, blocking αvβ3 integrins inhibited VWF-mediated melanoma cell adhesion [50]. MDA-MB-231 breast cancer cells have been found to express αvβ3 integrin, which can mediate VWF binding to tumour cells [50, 52, 54]. However, in this case, adhesion of VWF via the αvβ3 integrin induces apoptosis in breast cancer cells [18, 30]. The role of αvβ3 integrin in VWF-mediated apoptosis and metastasis is discussed in further detail in later sections of this review.

Taken together, this highlights several distinct mechanisms through which VWF may interact with breast cancer cells, by direct adhesion via a number of integrins or indirectly through platelet-VWF interactions.

## Elevated levels of VWF in patients with metastatic breast cancer

VWF has been shown to play an important role in tumour progression and metastasis [9]. Elevated levels of VWF in the plasma have been reported in various cancers, including breast, bladder, prostate and ovarian carcinoma, compared to benign disease and normal healthy controls [55], [56], [57], [58]. Moreover, studies have detected higher levels of plasma VWF in metastatic disease compared to primary cancer presentations [58], [59], [60]. Importantly, the increased plasma levels of VWF in patients with cancer have also been shown to be associated with a poorer prognosis [61]. In breast cancer specifically, an association has been found between an increase in VWF concentration and a higher tumour grade, and that VWF could potentially be a biomarker of relapse [62]. Notably, a significant rise in the plasma levels of VWF has been demonstrated in patients with malignant disease compared to benign conditions and healthy controls (*p*<0.005), with an even greater increase seen in patients with disseminated disease compared to early stage cancer (*p*<0.0001) [58]. The increased plasma VWF in patients with cancer is conventionally thought to originate from the activated endothelial cells and platelets [20]. However, emerging evidence suggests that some cancer cells, for example the gastric adenocarcinoma and osteosarcoma cells, also express VWF [20, 63].

In a study using a 4T1 murine model of breast cancer metastasis, plasma levels of VWF have been found to be significantly elevated at the late phases of metastasis, specifically in the fourth to fifth week after cancer cell inoculation, when robust metastatic lesions had formed in the lungs [64]. No significant changes were observed in the plasma levels of VWF at the early phases of metastasis, specifically in the first and second week after tumour cell inoculation in the 4T1 murine model, when only micrometastases were detected. This finding supports a role for VWF in cancer dissemination and the initiation of a metastatic focus formation [30, 65]. Intriguingly, VWF levels fell in the fifth week following inoculation of breast cancer cell in mice, however the underlying biological mechanism mediating this decrease remains unclear [64]. In addition, it has also been found that VWF levels increased within the primary tumour microenvironment, but not at the distal metastatic site [64]. This potentially indicates that the primary tumour and the associated microenvironment drive the progressive increase of VWF in the plasma, which also correlate with cancer progression with time [64].

It has been shown that VWF levels are strongly correlated with the protein levels of scatter factor (*p*<0.0001). This invasogenic and angiogenic cytokine is encoded by the MET oncogene, which is often aberrantly expressed in cancer pathologies [66]. Importantly, scatter factor is also associated with breast tumour aggressiveness [67]. Similarly, elevated VWF levels correlated with increased breast tumour invasiveness [67]. In support of this, clinical studies have reported markedly elevated serum levels of VWF in breast cancer patients with more aggressive disease stage (TNM of T2) (TNM is a cancer staging system - tumour (T), node (N) and metastasis (M)) compared to those with less aggressive disease stage (TNM of T1) (*p* = 0.019). Patients with advanced disease (TNM of M1) also had significantly higher levels of VWF than patients with less aggressive disease (TNM of T1) (*p* = 0.001) [68]. The elevated levels of VWF have also been correlated with higher levels of the cancer antigen CA15–3, a breast tumour marker which is also raised in disseminated disease (*p* = 0.027) [68].

## VWF as a regulator of breast cancer metastasis

Independent of its contribution to haemostasis, accumulating evidence suggests that VWF may also play several important roles in cancer metastasis [18]. For example, VWF may orchestrate cancer dissemination via an array of pathways, including angiogenesis and hypercoagulopathy [54,69,70].

### Angiogenesis

One hallmark of cancer is angiogenesis, which promotes the proliferation, invasion and migration of cancer cells. It is a complex multistep process involving an angiogenic switch to allow vascular proliferation and cancer progression when the tumour grows to a certain size, where the oxygen and nutrient requirements can no longer be met [70]. Potent angiogenic factors include vascular endothelial growth factor (VEGF) and fibroblast growth factor 2 (FGF-2), which are often present in a tumour microenvironment. Importantly, these factors have been shown to have a synergistic effect on the upregulation of VWF mRNA and protein levels in endothelial cells [70]. It has been reported that breast cancer cells exert a significant effect on the upregulation of angiogenic genes, including VWF, thus promoting metastasis [69]. Notably, this feature is limited to certain types of cancers. Specifically, breast and colon cancer cells are able to enhance the angiogenic properties of the endothelial cells, whereas osteosarcoma or rhabdomyosarcoma cells do not affect these angiogenic genes [69].

### Hypercoagulopathy and tumour cell-induced platelet aggregation

It has been demonstrated that patients with disseminated disease, including metastatic breast cancer, have a deficiency of VWF-cleaving ADAMTS-13 protease activity [54, 71, 72]. Interestingly, patients with metastatic disease have 165% more ultra-large VWF compared to patients with localised tumours (*p*<0.001) [72]. This observation may be attributed to a deficiency or dysfunction of ADAMTS-13 activity detected in the plasma of patients with metastatic cancer, or the augmented VWF secretion from the tumour microenvironment [54, 72]. Importantly, it has been demonstrated that this highly polymeric VWF has a significantly enhanced functionality evidenced by the ristocetin cofactor and tumour-induced platelet aggregation assays [72]. It has also been shown that VWF multimers with the largest size have a greater binding affinity to its platelet receptors, GPIbα and GPIIb/IIIa receptors under conditions of shear stress [73]. In a non-cancer setting, the dysfunctional ADAMTS-13 in patients with thrombotic thrombocytopenic purpura (TTP), results in the presence of highly adhesive ultra-large VWF multimers in the blood that bind tightly to platelets to form aggregates [74].

**Fig. 2:**
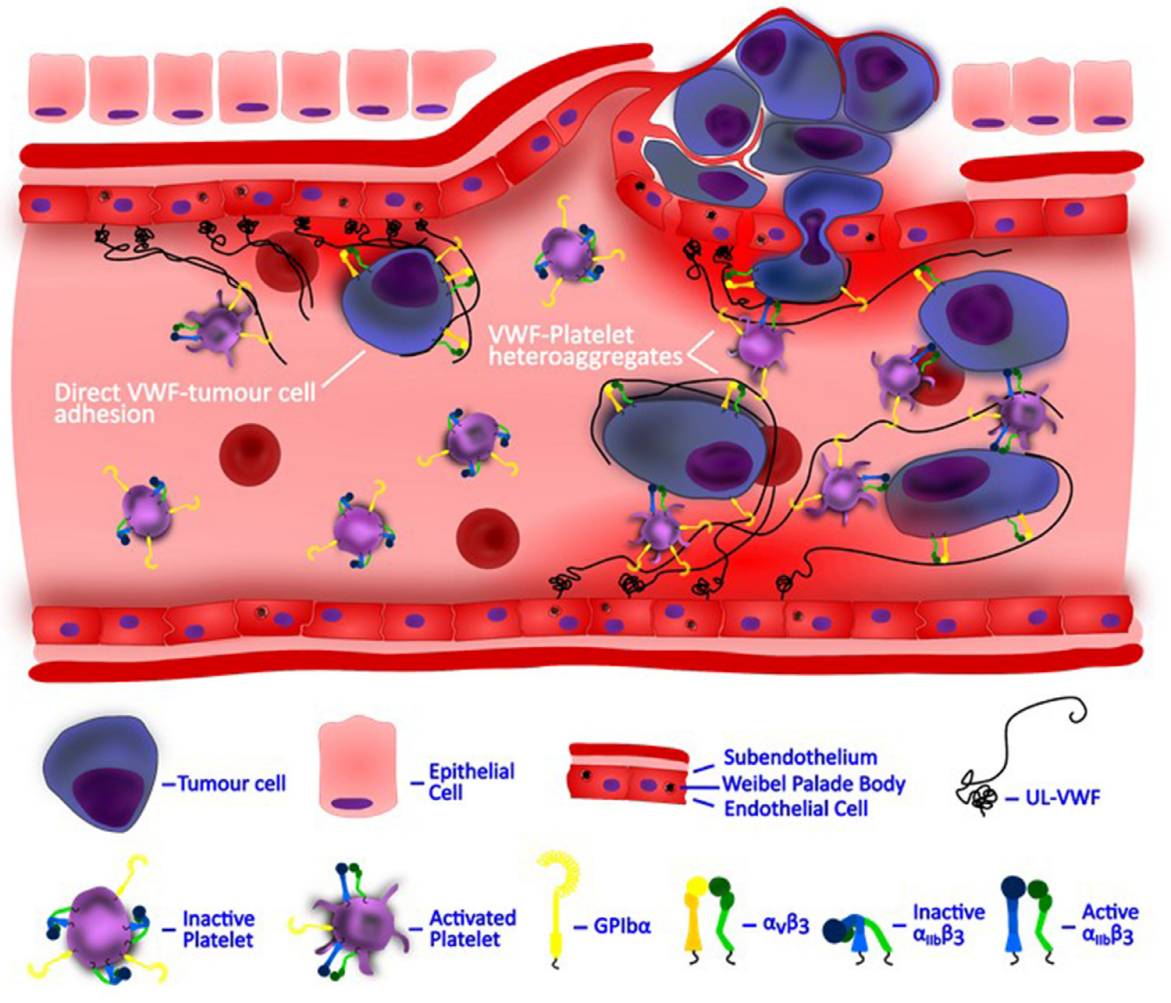
**Heterotypic aggregates comprising platelets, tumour cells and VWF.** The ultra-large VWF tethered along the endothelium mediates platelet adhesion and aggregation via GPIbα and α_IIb_β_3_ (GPIIb/IIIa) platelet receptors. Platelet-decorated VWF multimers may also tether tumour cells via the GPIIb/IIIa receptor and αvβ3 integrin. The resultant heterotypic aggregates formed along the endothelial surfaces may facilitate the extravasation of cancer cells across the endothelial cell wall and contribute to metastasis [50, 52, 54, 58, 72].

The increased concentration of the highly adhesive VWF multimers may modulate platelet-tumour cell interactions along the endothelium, contributing to tumour invasion and metastasis [72]. Platelets are well known to play an important role in metastasis. Cancer cells can cause aggregation of platelets in a process called tumour cell-induced platelet aggregation, which correlates to greater metastatic potential of the tumour cells [75, 76]. Consistent with this, the inhibition of this aggregation process decreases the metastatic potential of cancer cells without affecting the growth of the primary tumour [77, 78]. Platelet aggregates promote transmigration of tumour cells through the vessel wall via endothelial activation. In this process, VWF is important in potentiating the cancer-cell platelet aggregation [79]. On the one hand, VWF binds to platelets via the GPIbα and GPIIb/IIIa receptors, and activates the endothelium to increase vascular permeability. On the other hand, VWF binds to tumour cells via GPIIb/IIIa receptor or its hemi-identical twin αvβ3 integrin, thereby facilitating the extravasation of cancer cells through the activated endothelium [50, 52, 54]. Collectively, the ultra-large VWF binds to the platelet GPIbα and GPIIb/IIIa receptors, and at the same time adheres to tumour cells via GPIIb/IIIa receptor and αvβ3 integrin, to facilitate the metastatic process (Fig. 2). The resultant heterotypic aggregates are more likely to adhere to endothelial surfaces compared to single tumour cells [58, 72]. In addition, it has been found that the release of tumour thrombin induces the production of VWF and facilitates the adhesion of cancer cells to the endothelium [80, 81]. In support of this, many in vivo studies that utilise antibody treatment have also shown marked reduction of metastatic potential in cancer cells following the inhibition of GPIbα and GPIIb/IIIa receptor sites and VWF [72, 80, 82, 83].

### Shielding metastatic cells from chemotherapy

VWF has also been demonstrated to protect disseminated tumour cells (DTCs) from chemotherapy [84]. In one study, bone marrow mesenchymal stem cells and microvascular niches were seeded with basal HMT-3522-T4–2 breast tumour cells to mimic DTCs. Following chemotherapeutic treatment with doxorubicin, VWF knockdown resulted in apoptosis of up to 70% of DTCs, and the level of apoptosis correlated with the level of VWF depletion. In fact, the levels of apoptosis were similar to those treated with an antibody that inhibited the function of αvβ3 integrin. This suggests that the αvβ3 integrin protects DTCs from chemotherapy through downstream signalling triggered by VWF, although the exact underlying mechanism remains unclear [84]. Notably, the depletion of VWF did not affect the survival or outgrowth of the breast tumour cells in the absence of chemotherapy [84]. Importantly, inhibiting the integrin-mediated interactions between DTCs and the perivascular niche, driven partly by VWF, sensitises DTCs to chemotherapy [84].

### The paradoxical role of VWF in breast cancer metastasis

Some studies have shown a protective role of VWF in the initiation of metastatic foci. The adhesion of circulating VWF to tumour cells via αvβ3 integrin, mediates apoptosis of several tumour cell lines in vitro, including breast cancer cells MCF-7. Mechanistically, the apoptotic process occurs via the downstream signalling of the TP53 phosphorylation and CASP3 activation pathways [30]. In this case, ADAM28 cleaves and inactivates VWF, inhibiting the process of apoptosis, thus promoting lung metastasis [30]. Interestingly, certain aggressive cancer cells are not susceptible to the pro-apoptotic function of VWF. This resistance of VWF-mediated apoptosis was dependent on tumour cell expression of a specific metalloproteinase ADAM28 that cleaves VWF, rendering its apoptotic function inactive. The aggressive MDA-MB-231 breast cancer cells have been shown to express higher levels of ADAM28 and demonstrate resistance to VWF-induced apoptosis, whereas the less aggressive MCF7 breast cancer cells have been found to express lower levels of ADAM28 and are susceptible to apoptosis [30, 85]. Importantly, the knockdown of ADAM28 in the MDA-MB-231 cells resulted in increased programmed cell death and decreased lung metastases. This suggests that tumour-expressed ADAM28 inactivates VWF within the circulation, potentially favouring tumour cell survival within the vasculature, thus promoting cancer dissemination. Notably, the VWF-mediated apoptotic effect appears to be specific to tumour cells, as VWF did not induce cell death in non-neoplastic cell lines examined. This highlighted the high specificity with which VWF induces tumour cell apoptosis [30, 42].

Similar findings have also been observed in vivo in other types of cancer. Specifically, using a VWF-deficient murine model following the injection of B16-BL6 murine melanoma cells or Lewis lung carcinoma (LLC) cells, VWF was found to play a protective role against tumour cell dissemination by inducing apoptosis of metastatic cells [18]. However, it is noteworthy that previous studies have demonstrated that the inhibition of VWF using monoclonal antibodies prevented metastasis formation in mice [86]. This is not the first time that contradicting results have been reported from genetically-altered mice compared to inhibition studies using pharmacological agents. The opposing results in these VWF studies could potentially be explained by the genetic ablation of VWF in the VWF-deficient mice, as opposed to the partial and transient inhibition of VWF in antibody studies, in which only the VWF plasma compartment is targeted [18].

While ADAM28 acts as a semi-functional homologue of ADAMTS-13 in cleaving circulating VWF, it is interesting to note that while ADAMTS-13 levels have been shown to decrease in a range of metastatic cancer including breast, ADAM28 expression is correlated with advanced disseminated disease [30, 72, 87, 88]. This is potentially due to the localised, pathological expression of ADAM28 on tumour cells. Conversely, ADAMTS-13 circulates as a soluble protease cleaving VWF multimers within the circulation under physiological conditions. Collectively, it is interesting to speculate that the VWF-apoptosis axis may be specific to tissue localisation and microenvironment.

## Therapeutic implications of VWF in breast cancer metastasis

### HDAC inhibitors

Histone deacetylase (HDAC) inhibitors are a class of anti-cancer agents that induce apoptosis and cell cycle arrest in tumour cells [89]. HDAC 1 and 2 can act as promoters or repressors of the VWF gene in a cell type-specific manner [90, 91]. Specifically, the recruitment of HDAC, histone acetyltransferase (HAT) and GATA6 trans-acting factor to the VWF promoter region, determines the activation or repression of the VWF gene [90]. In endothelial cells, the HDAC, nuclear transcription factor Y (NFY) and GATA6 interaction is shifted towards the favouring of the VWF gene promoter activation, potentially through the endothelial cell-specific signalling [90]. In non-endothelial cells, however, the NFY inhibits the activation of the VWF promoter region via HDAC recruitment [90]. An in vivo study of breast carcinoma in a murine model treated with the HDAC inhibitor MS-275 demonstrated significantly reduced tumour growth, decreased VWF-positive blood vessels (decreased angiogenesis), decreased lung metastasis and reversed epithelial-mesenchymal transition (EMT) [92]. Mechanistically in the tumour cells, the HDAC inhibitors enhanced the apoptosis-inducing potential of tumour necrosis factor-related apoptosis-inducing ligand (TRAIL) [92, 93]. In addition, these data suggest that transcriptional targeting of VWF expression via HDAC inhibitors may serve to attenuate breast cancer metastasis.

### Desmopressin

Clinically, it has been suggested that surgery induces shedding of tumour cells into the circulation or lymphatic system, posing an increased risk for the accelerated process of micrometastatic disease during the perioperative period [94]. The interruption of this process might minimise the survival of tumour cells and thus reduce the potential formation of metastatic foci from the dislodged cancer cells. Based on previous studies that have shown a protective role of VWF in the initiation of metastatic foci, a phase II dose-escalation clinical trial (NCT01606072) investigated the provision of high-dose perioperative desmopressin (dDAVP) to reduce metastasis in breast cancer patients [95]. Functionally, dDAVP increases plasma levels of VWF, coagulation factor VIII (FVIII) and tissue plasminogen activator (t-PA) [96]. In a non-cancer setting, dDAVP is the treatment of choice in patients with von Willebrand disease (VWD; a genetic disorder caused by reduced or dysfunctional VWF) to stimulate the release of endogenous VWF into the plasma [97]. Results from this clinical trial reported that high doses of perioperative dDAVP inhibited lymph node and early blood-borne metastasis in patients. This effect is potentially mediated by the enhanced endothelial VWF secretion with consequent haemostatic and antimetastatic effects [95].

## Conclusion

The interplay between the blood coagulation system and cancer dissemination has sparked an interest among researchers to further investigate the complex process of metastasis. Importantly, the mechanisms by which VWF may mediate metastasis in breast cancer are beginning to be elucidated, as implicated by several studies. VWF contributes to angiogenesis which enhances the dissemination of breast tumour cells to distal secondary sites [69, 70]. In addition, it has been shown that patients with metastatic breast cancer have reduced ADAMTS-13 protease activity, resulting in the presence of adhesive large VWF multimers in the plasma [71, 72]. The ultra-large VWF is capable of binding to cancer cells and platelets with high affinity, forming heterotypic aggregates that promote the adhesion to vessel walls and the subsequent transmigration of tumour cells across the blood vessel [72, 80, 81]. In addition, it has also been found that VWF shields the metastatic breast cells from chemotherapy-induced apoptosis [84]. Paradoxically, some studies have demonstrated the protective roles of VWF in metastasis [18, 30, 92]. As discussed in the previous section, the contradicting results could potentially be explained by the genetic ablation of VWF in both tissue beds and vasculatures in the VWF-deficient mice studies, in contrast to partial antibody-mediated depletion of circulating VWF [18]. These conflicting opinions also highlight the need for further research in the area in order to fully define the role of VWF in breast cancer metastasis. Clinically, HDAC inhibitors and dDAVP are potential therapeutic agents of interest which may aid in reducing the dissemination of breast cancer cells [92, 95]. However, further studies are warranted to confirm these findings, and to unravel their potential role as anti-metastatic agents in breast cancer.

In conclusion, VWF has a well described role in haemostasis, tethering circulating platelets along the endothelial cell wall in response to vascular injury. More recently however, novel biological roles for VWF have been reported including in inflammation, angiogenesis and cancer cell biology. For example, in breast cancer, plasma VWF levels are significantly elevated in patients with malignant disease compared to benign conditions and healthy controls. Importantly, these high VWF levels correlate with presence of metastatic disease and poorer prognosis. Moreover, elevated plasma VWF levels are an independent predictor of venous thromboembolism in cancer patients. Consequently, understanding the role of VWF in the setting of breast cancer may not only serve to attenuate metastasis but also reduce the risk of thrombosis. For the first time, this review systematically and specifically reports on the accumulating evidence for the biological role of VWF in breast cancer including interaction with breast tumour cells, apoptosis, angiogenesis and breast cancer metastasis.

## CRediT authorship contribution statement

**Chia Yin Goh:** Conceptualization, Writing – original draft, Visualization. **Sean Patmore:** Writing – original draft, Visualization. **Albert Smolenski:** Writing – review & editing. **Jane Howard:** Writing – review & editing. **Shane Evans:** Writing – review & editing. **Jamie O'Sullivan:** Writing – review & editing, Supervision. **Amanda McCann:** Resources, Writing – review & editing, Supervision, Funding acquisition.

## Declaration of Competing Interest

The authors have no conflicts of interest to disclose.
